# Process Optimization for Robotic Ultrasonic Strengthening of Aviation Blade Surfaces Based on Intelligent Compliance Control

**DOI:** 10.3390/mi14101920

**Published:** 2023-10-10

**Authors:** Shanxiang Fang, Yukai Zhu, Qinjian Zhang, Yong Zhang

**Affiliations:** 1 Department of Strategic and Advanced Interdisciplinary Research, Peng Cheng Laboratory, Shenzhen 518055, China; fangshx@pcl.ac.cn (S.F.);; 2 Department of Mechanical Engineering, National University of Singapore, Singapore 117575, Singapore; 3 School of Astronautics, Beihang University, Beijing 100191, China; 4 Mechanical Electrical Engineering School, Beijing Information Science & Technology University, Beijing 100192, China

**Keywords:** ultrasonic strengthening, robotic compliance control, aviation blade, surface quality, parameter optimization

## Abstract

In order to enhance the automation level and achieve high precision in the ultrasonic strengthening of aviation blade surfaces, this study focuses on investigating the intelligent control strategy and optimizing the machining parameters for robotic ultrasonic surface strengthening. By designing an intelligent compliance control method, the end-effector can achieve the compliant output of contact force. The fuzzy PID control method is used to optimize the regulation performance of the compliant force control system. This compliance control strategy enables the optimization of the compliance device, effectively improving the static and dynamic characteristics of the compliance controller. Based on this, an experimental method (RSM) is designed to analyze the interaction effects of contact force, feed rate, and repetition times on the surface quality of the blade. The optimal combination of robotic strengthening parameters is determined, providing a practical reference for the application of robotic compliance control in the ultrasonic strengthening of aviation blade surfaces.

## 1. Introduction

Titanium alloy aviation blades are mainly used in the front fans and low-temperature compressor of the aero-engine. These blades can control the airflow velocity, increase the airflow pressure, and make fuel fully burn [1,2]. Their quality determines the performance of the aero-engine. At present, blade fatigue fracture is one of the main problems of aero-engine failure. Improving the surface quality of aviation blades and enhancing their fatigue strength is an effective method to prevent fatigue fracture [3,4]. Therefore, the surface strengthening of titanium alloy aviation blades is of great significance to improve the service life and work efficiency of the aero-engine and improve the flight safety of aircraft [5,6,7].

Ultrasonic surface strengthening technology has characteristics similar to shot peening and laser shock strengthening [8,9], which can greatly improve the ability of titanium alloy parts to resist fatigue crack initiation and propagation. In order to improve the automation level of ultrasonic strengthening and realize the high precision strengthening of the blade surface, it is necessary to apply an industrial robot with a strengthening device to complete the process automatically. The advantages of industrial robots in the field of automated processing are very prominent, for example, a robotic complete finishing process is introduced for automated robotic deburring applications with low machinability materials on aero-engine casings with complex geometries [10], thus enabling a reduction in process operating time and improving processing quality. In the process of ultrasonic strengthening of the blade surface, the contact force between the strengthening tool and the workpiece surface is very important for forming a residual stress layer [11,12]. The constant contact force can ensure the oscillation stability of the strengthening tool, which is a key factor in inducing elastic–plastic deformation of the workpiece material [13,14,15]. Therefore, compliance control of contact force is required in the process of ultrasonic strengthening of the blade surface to keep constant contact force between the strengthening tool and the area to be strengthened in the normal direction.

As the research on the control technology of robots has entered the intelligent stage, the intelligent force control algorithm of robots has also been developed accordingly. The object of intelligent control has the characteristics of an uncertain mathematical model, high nonlinearity, and complex task requirements, which can solve complex system control problems that are difficult to solve using traditional methods [16,17]. Fuzzy control is a kind of computer digital control technology based on fuzzy set theory, fuzzy variables, and fuzzy logic reasoning. It can be controlled by the operator’s control experience without establishing a mathematical model. It can be effectively applied to uncertain systems and has strong robustness [18,19]. To solve the problem of controlling a complex robot system with disturbances, Zheng et al. [20] design a double fuzzy sliding mode controller based on the combination of sliding mode control and fuzzy logic control. The proposed method is applied to a KUKA industrial robot system. Li et al. [21] design a fuzzy controller to improve system force tracking performance in highly dynamic unknown environments. Considering a new fuzzy sliding mode control of a 7-DoF upper-limb exoskeleton robot [22], fuzzy control is applied to improve the sliding mode controller, which is robust against external disturbances and unknown dynamics such as friction forces, different mechanisms mass, backlash, and input saturation. With the increasing demand for using robotic manipulators in industrial applications, controllers specific to performing repeatable tasks are required. These controllers must also be robust to model uncertainties. Yilmaz et al. [23] introduce a repetitive learning control method fused with adaptive fuzzy logic techniques. Specifically, modeling uncertainties are first modeled with a fuzzy logic network, and an adaptive fuzzy logic strategy with online tuning is designed. Huynh et al. [24] propose integrating impedance control, fuzzy logic control, and iterative learning control into a unified control schema for a robot gripper. A fuzzy impedance controller is developed to dynamically estimate optimal impedance parameters during the gripping of unfamiliar objects. Additionally, iterative learning control is used to enhance the sample dataset, thereby improving the design of the fuzzy rule base.

Analyzing force control in the robotic ultrasonic surface strengthening process, the compliance control needs to solve the problem of maintaining constant contact force under multiple impacts and vibration conditions. By establishing a dynamic relationship between the contact force and the robot end-position deviation, the robot can maintain the appropriate output force in a constrained environment.

In addition, under the condition that the strengthening path of the aviation blade surface is already planned, the quality of robotic surface strengthening is also affected by process factors referred to in other research on robotic surface processing, such as robotic grinding, robotic milling, and robotic polishing. Joshi et al. [25] develop a process model for robot–workpiece interactions and grinding process cycle time. For a given robot stiffness, minimizing the face grinding cycle time requires the process to be operated at the highest achievable wheel speed and feed rate with a wheel of higher hardness. For robotic milling processes, robots should have to execute highly dynamic and accurate force control. Zaeh et al. [26] present a strategy of machine–process interaction with online adaptation mechanisms for increased system robustness, which can solve the problem of external static and dynamic process forces leading to static deflections and dynamic excitations. Jiang et al. [27] conduct robot-assisted milling experiments based on the theoretical dynamic model for the milling process. The proposed model can successfully estimate the milling force and the cutting depth in experimental conditions. The approach can successfully predict the current milling status and is sensitive to changes in force conditions. Gaz et al. [28] propose a control algorithm that is able to distinguish the external torques acting at robot joints during robotic polishing operations. The controller can estimate joint torques due to contact forces/torques applied at any place along the robot structure online. Ochoa et al. [29] present a computed torque impedance control architecture for robotic-assisted mold polishing. A Cartesian impedance control referred to as the end-effector frame with posture optimization is designed to analyze the position and force patterns. Analyzing the above research, robotic ultrasonic strengthening of the aviation blade surface can be considered a processing method with multi-process fusion. The main factors affecting the strengthening quality are contact force, feed rate, and repetition times. Subtle adjustments to each of these three factors can have a significant impact on the surface quality after strengthening, so optimization of processing parameters before strengthening is an indispensable and important part.

For the demand for automatic ultrasonic strengthening of aviation blade surfaces, this paper proposes a compliance control method for robotic ultrasonic surface strengthening. The innovative work mainly includes (1) fuzzy PID control is used to control the output force of the end-effector to improve the dynamic regulation performance and tracking robustness of the system, so as to maintain constant contact force between the ultrasonic tool and the blade surface under multiple impacts and vibration conditions and (2) an optimization experiment is designed to study the interaction influence of the robot machining parameters on the surface quality, and the optimal combinations of processing parameters are obtained. This study provides a new technical solution for the surface strengthening of aviation blades and provides a feasible reference for practical application.

## 2. Compliant Control of Robotic Ultrasonic Surface Strengthening

**Figure 1:**
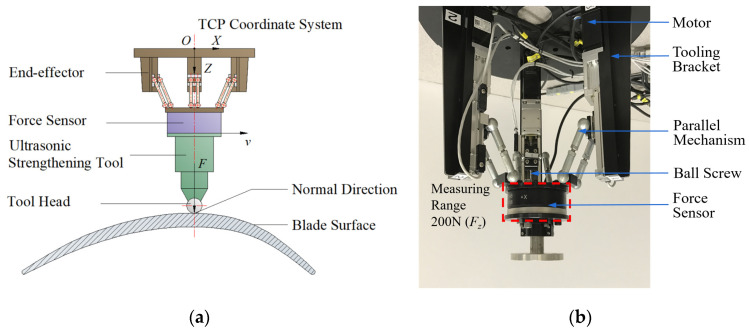
Schematic diagram of ultrasonic strengthening of the blade surface. (**a**) Schematic diagram of ultrasonic surface strengthening. (**b**) The end-effector.

In the process of robot ultrasonic surface strengthening, the contact force applied by the strengthening tool on the workpiece surface is crucial for the formation of residual stress layers. The contact force not only ensures the stability of the strengthening tool vibration but also serves as a key factor in inducing elastic–plastic deformation of the workpiece material. Therefore, during the feed motion of the strengthening tool along the planned path, it is necessary to maintain a constant normal contact pressure between the tool head and the strengthening area, as shown in Figure 1a. This implies that the output force in the *Z*-axis direction of the TCP coordinate system needs to be controlled during the strengthening process. However, in the interaction process between the ultrasonic strengthening tool and the aviation blade surface, due to the influence of the dynamic environment, the geometric path of the tool will be constrained. The error of calibration and trajectory planning may cause the tool to deviate from the target trajectory, resulting in a change in the contact force. The rigidity of industrial robots, blade workpieces, and the working environment is relatively large. In order to obtain the desired output contact force and tool pose, the control model needs to be very accurate [30], but this is difficult to achieve in actual control tasks. The coupling of force control also makes the control system more complex. In addition, the coupling of pose control and force control makes the control system more complicated.

This study validates the compliant force control method using an end-effector (mini manipulator) [31], as illustrated in Figure 1b. During the robot-assisted ultrasonic surface strengthening process, separate control over the position and force at the end of the strengthening tool is achieved without any mutual constraints. Position control is achieved with the KUKA KR60 robot, while force control is implemented by the end-effector. This decentralized control method makes the force/position control of the robotic ultrasonic strengthening system easier to implement. The output torque of the motors can be converted into the output force using the ball screw and parallel mechanism. By adjusting the input voltage signal, the end-effector can be driven to output the constant Z-direction pressure during the processing. There are many nonlinear factors in the control model of the device, and it is hard to quantify accurately. In order to control the end-effector, its experimental measurement model is established.

The tool head for ultrasonic surface strengthening is a spherical ball that rolls on the blade surface in the machining direction and is lubricated with oil during the strengthening process, resulting in very little friction. Therefore, the influence of friction force can be properly ignored in the mechanical model of ultrasonic surface strengthening. It only needs to consider the control of the output force in the *Z*-direction of the end-effector.

The force balance equation of the end-effector is established as Equation (1). Ignoring the friction of the ball screw, the Laplace transform of Equation (1) can be expressed as Equation (2).

(1) $$Mk-F_{f}=G\frac{d^{2}y}{dt^{2}}+C_{p}\frac{dy}{dt}+F_{n}$$

(2) $$M\left(s\right)k=Gs^{2}Y\left(s\right)+C_{p}sY\left(s\right)+F_{n}\left(s\right)$$

where *M* is the output torque, *k* is the proportionality coefficient, *F_f_* is the friction force, *G* is the total mass of the moving parts, *y* is the moving component’s displacement, *C_p_* is the frictional damping coefficient, and *F_n_* is the output force.

Considering the motor parameters and force output relationship, the transfer function of the end-effector system can be derived as Equation (3). It shows the relationship between input control quantity and output contact force.

(3) $$G\left(s\right)=\frac{M\left(s\right)}{U\left(s\right)}\cdot \frac{F\left(s\right)}{M\left(s\right)}=\frac{K_{m}}{T_{s}s+1}\cdot \frac{K_{e}k}{Gs^{2}+C_{p}s+K_{e}}=\frac{K_{e}K_{m}k}{\left(Gs^{2}+C_{p}s+K_{e}\right)\left(T_{s}s+1\right)}$$

where *U* is the input voltage, *K_e_* is the equivalent stiffness coefficient, *T_s_* is the inertia time coefficient of the motor, and *K_m_* is the forward gain.

The fuzzy PID control method is used in this paper, which does not require an accurate system control model, so the time domain measurement method is used to measure the model. The experimental measurement model is calculated as Equation (4).

(4) $$G\left(s\right)=\frac{11.374}{0.00182s^{3}+0.045225s^{2}+0.33s+1}$$

**Figure 2:**
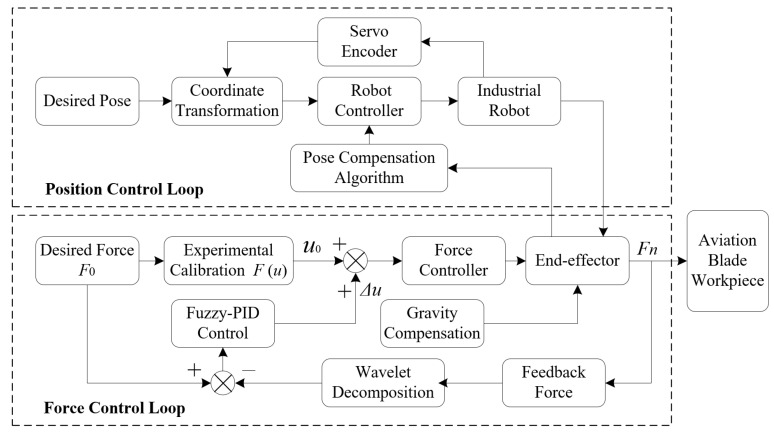
Force/position control of robotic ultrasonic surface strengthening.

Figure 2 is a block diagram of force/position control of robotic ultrasonic surface strengthening. In the force control loop, the force control signal is output by the force controller to the compliant force control device (end-effector), thereby achieving the adjustment of the output contact force.

## 3. Research on Intelligent Compliance Contact Force Control

### 3.1. Fuzzy PID Control of Contact Force

In the process of robotic ultrasonic surface strengthening, when the robot runs fast or the curvature of the strengthened area changes greatly, the position and posture of the tool will change significantly in a short time, which puts forward relatively high requirements on the output response speed of the end-effector. For the ultrasonic strengthening process of the blade surface, the multi-impact and vibration environment also requires the end-effector’s controller to have good control stability and steady-state accuracy.

Fuzzy control has strong parameter adaptability and anti-interference ability, which can make the system respond quickly and effectively suppress the overshoot of the system. However, inevitable steady-state error will be produced by fuzzy control. The integral link of the classical PID controller can eliminate the steady-state error and make the system track the target value without static error [32]. Therefore, the advantages of PID control and the fuzzy control algorithm can be combined, so that the controller can adapt to the parameter changes in the controlled object. The control structure will be relatively simple, which will help to realize the application of specific engineering tasks.

**Figure 3:**
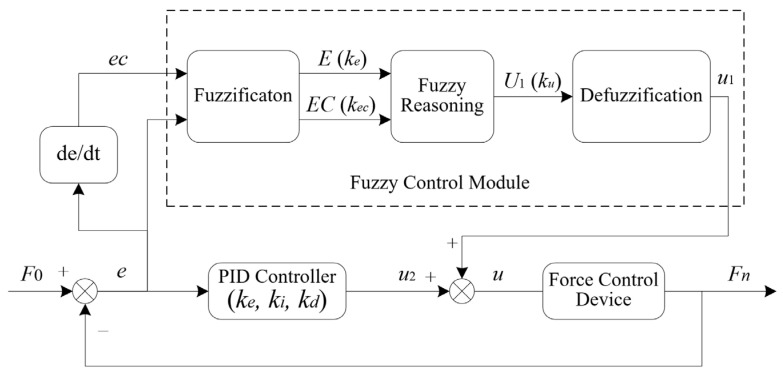
Block diagram of fuzzy PID control system.

Figure 3 indicates the parallel fuzzy PID control method, in which fuzzy control and PID control are completely juxtaposed with each other to control the system in a parallel way. According to the given input and feedback signals, the fuzzy controller calculates the deviation *e* and the current deviation variation rate *ec* and then makes fuzzy inferences using fuzzy rules. At the same time, the PID control method is used to adjust the control system. The output values of fuzzy control and PID control are combined to adjust the output value of the system. This parallel control method can not only avoid the steady-state error caused by fuzzy control but also improve the deficiency of PID control in an unsteady system to a large extent. Compared with the conventional serial fuzzy PID control method, this algorithm is easier to implement and more suitable for engineering practice. Therefore, the parallel fuzzy PID control method is selected to control the output contact force of the end-effector.

### 3.2. Fuzzy Controller Design

**Figure 4:**
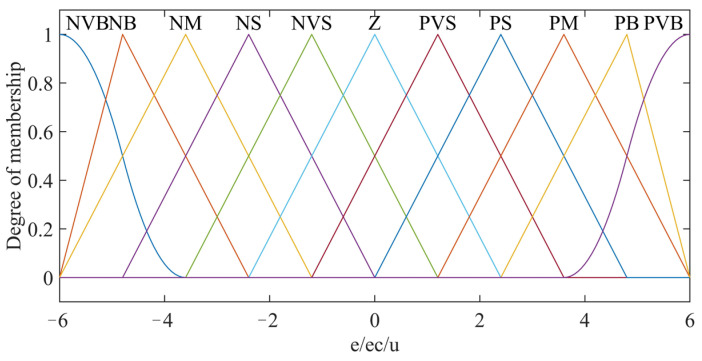
The membership functions of *e*/*ec*/*u*.

A fuzzy controller of contact force is designed. The input deviation *e*, deviation variation rate *ec*, and output *u* of the end-effector’s system are, respectively, set to 11 fuzzy subsets as {NVB, NB, NM, NS, NVS, ZO, PVS, PS, PM, PB, PVB}, that mean {negative very big, negative big, negative middle, negative small, negative very small, zero, positive very small, positive small, positive middle, positive big, positive very big}. The domain of discourse can be set to [−6, 6]. Considering the coverage and sensitivity of the domain, the fuzzy subsets adopt a triangular membership function, S-shaped membership function, and Z-shaped membership function, and the membership functions of *e*, *ec,* and *u* are shown in Figure 4.

The respective quantization factors of the system input value *e*, *ec*, and output value *u* can be initially set as Equation (5).

(5) $$k_{e}=\frac{2n}{b_{e}-a_{e}},k_{ec}=\frac{2n}{b_{ec}-a_{ec}},k_{u}=\frac{2n}{b_{u}-a_{u}}$$

where *a_e_* and *b_e_* indicate the actual variation interval [*a_e_*, *b_e_*] of the input deviation *e*, *a_ec_* and *b_ec_* indicate the actual variation interval [*a_ec_*, *b_ec_*] of the input deviation variation rate *ec*, *a_u_* and *b_u_* indicate the actual variation interval [*a_u_*, *b_u_*] of the system output value *u*, and *n* is their domain of discourse.

The control quantity of the fuzzy controller should be adjusted according to the following principles:

(1). When the deviation (e) is relatively large, irrespective of the value of the error change (ec), the control quantity should be increased to eliminate the error.
(2). When the deviation (*e*) is relatively small, to avoid excessive overshooting and to rapidly approach a steady state, the value of the deviation variation rate (ec) should be considered to adjust the control signal. If *ec* is positive, indicating an increasing trend in the deviation, the control quantity should be increased to prevent further error growth. If *ec* is negative, indicating a decreasing trend in the deviation, the control quantity can be set to a lower value, allowing the output signal to approach the desired setpoint.

According to the adjustment principle of control quantity, the fuzzy control rule table of contact force is established, as shown in Table 1.

There are 121 fuzzy rules in Table 1, and the corresponding fuzzy rule statements are as follows:

(1) If (*e* is NVB) and (*ec* is NVB), then (*u* is NVB);
(2) If (*e* is NVB) and (*ec* is NB), then (*u* is NVB);

··· ··· ···

(120) If (*e* is PVB) and (*ec* is PB), then (*u* is PVB);
(121) If (*e* is PVB) and (*ec* is PVB), then (*u* is PVB).

The relation between each fuzzy statement is “or”, and the fuzzy set *U* of the output is described as Equation (6).

(6) $$U=u_{1}+u_{2}+u_{3}+\cdot \cdot \cdot +u_{121}$$

In order to obtain the exact quantity, the fuzzy quantity is clarified using the method of defuzzification. The barycenter method (weighted mean method) is applied to solve the fuzzy quantity, shown in Equation (7). The fuzzy set membership degree *μ* of the output is taken as the weighting coefficient. Specifically, the product of each element *x_i_* (*i* = 1, 2, ···, 11) and its corresponding *μ*(*i*) is summed, and then the mean *x*_0_ is obtained.

(7) $$x_{0}=\frac{\sum_{i=1}^{11}x_{i}\mu \left(i\right)}{\sum_{i=1}^{11}\mu \left(i\right)}$$

where *x*_0_ is the decision result of the fuzzy set obtained using the barycenter method, and the actual accurate value *u* of the control quantity can be obtained using Equation (8).

(8) $$u=k_{u}x_{0}$$

The quantization factors are initially set as *k_e_* = 0.21, *k_ec_* = 0.17, and *k_u_* = 2.0 according to Equation (5). In the subsequent control simulation, the quantization factor can be changed near this value, and then the dynamic performance of the system during the step response can be analyzed, including the overshoot, response time, and steady-state error.

### 3.3. Fuzzy PID Control Simulation of Contact Force

**Figure 5:**
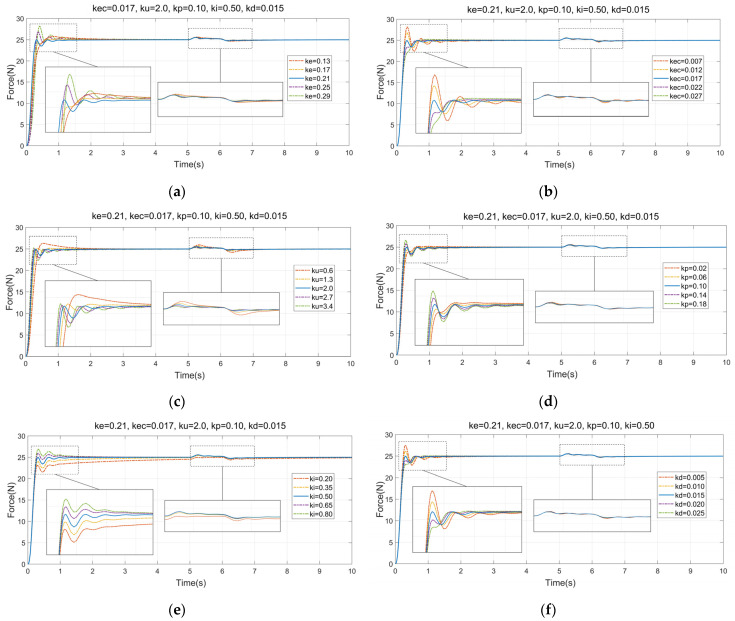
The effect of the quantization factors on the system response of fuzzy PID control. (**a**) The effect of *k_e_*; (**b**) the effect of *k_ec_*; (**c**) the effect of *k_u_*; (**d**) the effect of *k_p_*; (**e**) the effect of *k_i_*; and (**f**) the effect of *k_d_*.

In order to verify the influence of fuzzy PID control on the dynamic adjustment performance of the end-effector, it is necessary to carry out simulation research on the control algorithm. As shown in Figure 3, the adjustable quantization factors of the fuzzy control module are set as *k_e_*, *k_ec_*, and *k_u_* respectively, where *k_e_* is the quantization factor of the input deviation, *k_ec_* is the quantization factor of the deviation variation rate, and *k_u_* is the quantization factor of the output value. The quantization factors and PID control parameters *k_p_*, *k_i_*, and *k_d_* are jointly adjusted, and the influence of each parameter on the system response curve is analyzed. The step interference signal is added at the fifth second to test the anti-interference performance of the control algorithm. The simulation results are shown in Figure 5.

According to Figure 5a,d, it can be seen that the quantization factors *k_e_* and *k_p_* have a significant influence on the response speed and waveform of the system output. The larger the value of *k_p_*, the faster the response speed, but with the increase in *k_e_* and *k_p_*, the oscillation and overshoot of the system response curve will also be caused at the early stage of the regulation. According to Figure 5b,f, it can be seen that the overshoot of the system can be effectively suppressed by quantization factors *k_ec_* and *k_d_*, but it will prolong the time for the system to reach a steady state. In Figure 5c, *k_u_* is the quantization factor of output *u*, and it has a direct influence on the output of the whole system signal. This will not only affect the response speed of the system but also affect the early adjustment fluctuation shape of the system response curve, so the oscillation of the control system can be improved by adjusting *k_u_*. As shown in Figure 5e, the quantization factor *k_i_* is an integral coefficient, which significantly affects the speed at which the system output reaches the steady-state value. It shows that the steady-state error of the system can be eliminated by adjusting *k_i_*. For interference suppression, *k_u_* and *k_i_* have an obvious influence on the adjustment of interference. The control algorithm has good anti-interference performance, and the system can be adjusted effectively in a short time.

### 3.4. Experimental Verification of the Compliance Control Algorithm

**Figure 6:**
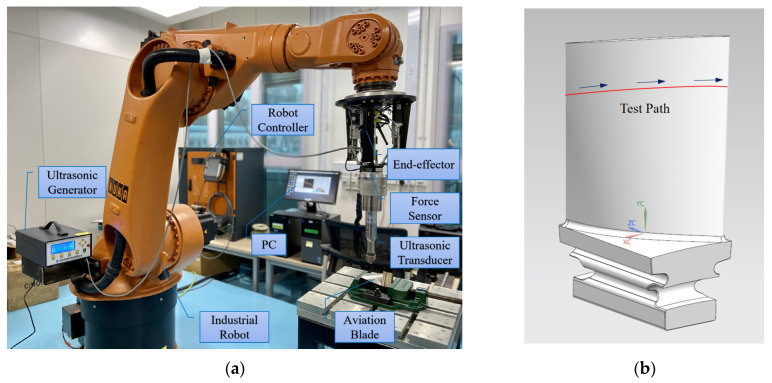
Output force control experiment of the end-effector. (**a**) Experimental platform. (**b**) Experimental trajectory.

An experimental platform for robotic surface ultrasonic strengthening is built, and one strengthening path on the blade surface is selected as the experimental trajectory. As shown in Figure 6, the output force control experiment of the end-effector is carried out. The performance of the compliant force control algorithm for dynamic adjustment of output force is studied, with the tool head pose changing during the robotic strengthening operation. The actual output force *F_n_* is measured with the force sensor (SRI M3704B) at the end of the end-effector; the steady-state error of the force sensor is ≤2% F.S.

**Figure 7:**
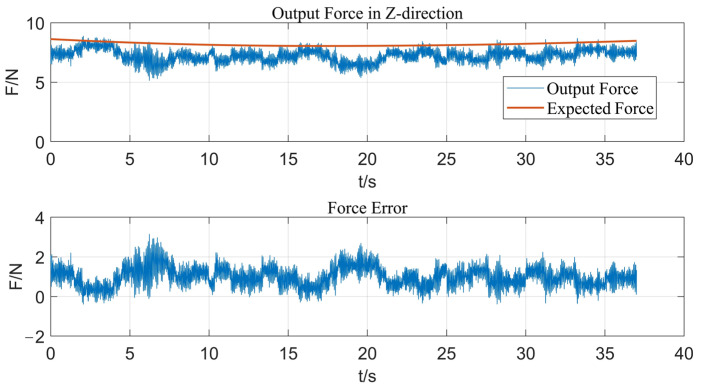
Response curve of output force in Z-direction with fuzzy control.

**Figure 8:**
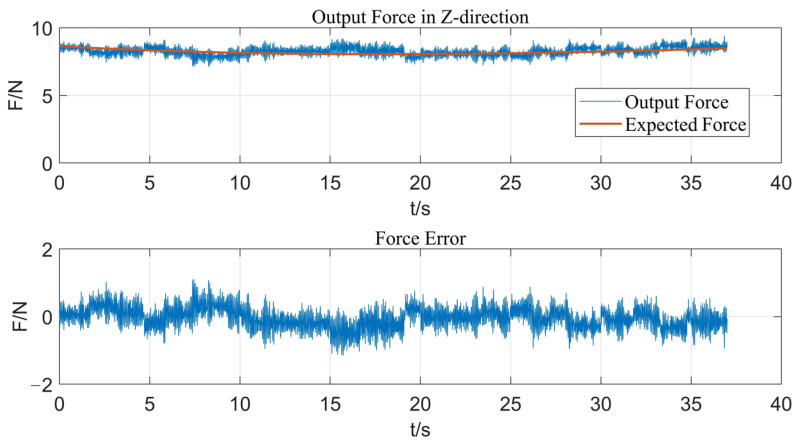
Response curve of output force in Z-direction with fuzzy PID control.

Figure 7 shows the output force response curve of fuzzy control when strengthening along this path. With the fuzzy control method, the response speed of the output force of the end-effector is relatively rapid, but there is always obvious steady-state error in the control process, and the output force error is in the range of 0–3 N, so the control effect cannot meet actual ultrasonic strengthening requirements. Then, fuzzy PID control is used, and the experimental result is shown in Figure 8. The actual output force can well follow the expected force, and the output force error is kept within ±1 N, which meets the control precision requirements and effectively eliminates the steady-state error caused by fuzzy control. This is because the control deviation of the system is usually large at the initial stage of the control process; therefore, the fuzzy control module can rely on its advantages of rapid adjustment to make the system quickly approach the target value with no overshoot or small overshoot and improve the dynamic characteristics of the system. When the deviation is small, the PID control module has the advantages of high control precision and small static error, which improves the steady-state precision of the system. Therefore, by adopting the parallel design of fuzzy control and PID control, the two algorithms will not interfere with each other, and each one will control according to the deviation between the feedback value and the target value of the current system based on their control laws. It effectively avoids the influence of unreasonable parameters of the former control algorithm on the performance of the latter control algorithm in serial control.

Therefore, parallel fuzzy PID control is an effective method for improving traditional PID control. It not only has the advantages of high control precision of traditional PID control but also has the advantages of fast, flexible, and strong adaptability of fuzzy control. The output performance of the end-effector has good dynamic characteristics and static characteristics, so as to meet the requirements of the robotic ultrasonic strengthening system for contact force control.

## 4. Optimization Experiment Study on the Machining Parameters of Robotic Ultrasonic Surface Strengthening

In order to obtain good machining quality (such as surface roughness, surface hardness, and residual compressive stress layer), it is necessary to optimize the machining parameters in the robotic ultrasonic surface strengthening process. Ultrasonic parameters (ultrasonic frequency and amplitude) and robot machining parameters (contact force, feed rate, and repetition times) mainly affect machining quality. The research on ultrasonic parameters of surface strengthening for titanium alloy is very mature and can be directly referred to. This has been described in a previous work about the path planning of robotic ultrasonic surface strengthening for turbine blades [13]. Robot application technology is the focus of this paper, so the effects of the robot machining parameters on the surface roughness and hardness of the blade workpiece after ultrasonic surface strengthening are studied in this section.

### 4.1. Experimental Scheme of the Response Surface Design Method

Conventional experimental design methods include factorial design, orthogonal design, uniform design, etc., but these experimental methods cannot intuitively discriminate the optimization region. Response surface methodology (RSM) is a more optimized statistical experimental design method. It can obtain the surface graph of the influence of various factors on the experimental results so that the optimized region in the experimental design can be obtained intuitively. In addition, the related experimental parameters can be comprehensively studied according to less experimental times using RSM [33,34]. The RSM model can be expressed as the following quadratic polynomial function [35,36]:

(9) $$y=\beta_{0}+\sum_{i=1}^{k}\beta_{i}x_{i}+\sum_{i=1}^{k}\beta_{ii}x_{i}^{2}+\sum_{i<j}^{k}\beta_{ij}x_{i}x_{j}+\varepsilon$$

where *y* is the response variable, *ε* is the normal random error, *β*_0_ is the regression intercept, and *β_i_*, *β_ii_*, and *β_ij_* are the regression coefficients.

Methods that work well in RSM include Box–Behnken Design (BBC) and Central Composite Design (CCD) methods [37,38]. The CCD method has good portability and orthogonality and has sequential properties. It can fit the surface better than BBC, and the error of the predicted value is smaller. The Central Composite Circumscribed design (CCC) of CCD has the best model, so the CCD-CCC method is selected for the experimental design.

Contact force, feed rate, and repetition times are selected as the factors affecting the robotic ultrasonic strengthening quality of the aviation blade surface. The center point (0, 0) of the CCD-CCC is used to determine the experimental error and reproducibility, (−1, 1) represents the low and high levels of variables, and (±*α*, 0, 0), (0, ±*α*, 0), and (0, 0, ±*α*) are axial points. *α* is the distance of the axial point from the center, *α* = 2*^k^*^/4^; *k* is the number of experimental factors, *k* = 3; so, *α* = 1.682. Taking the surface roughness reduction and surface hardness of the blade workpiece after strengthening as the response values, the codes and levels of CCD-CCC factors are shown in Table 2.

The robotic strengthening path is planned according to the surface curvature. The research on ultrasonic strengthening of TC4 titanium alloy is sufficient, and the ultrasonic parameters can be used for reference [13]. The parameters of the ultrasonic device in this experiment are set as an output amplitude of 30 μm; a frequency of 21.3 kHz; and an electric current of 2A. Appendix A Table A1 is the CCD-CCC table of the RSM design.

**Figure 9:**
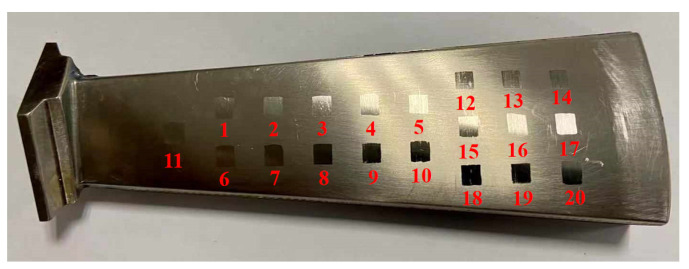
Experimental area division of RSM on the aviation blade surface.

The blade workpiece is machined using the milling process. Some studies have shown that precision milling can obtain good machining quality [39,40], but milling processes will create tensile stress on the blade surface. Ultrasonic surface strengthening can create a residual compressive stress layer on the blade surface, significantly improving fatigue resistance. Also, higher surface smoothness and surface hardness can be achieved. Considering the cost of relevant experiments, we first conduct experiments to study the effect of robotic strengthening parameters on surface roughness and surface hardness. Finally, residual stress testing is conducted on the surface that is strengthened with the optimized parameters. The blade surface is divided into 20 test areas, as shown in Figure 9. Each area is 5 mm × 5 mm in size and contains about 15 paths, which can effectively carry out the experimental verification of ultrasonic strengthening. According to the experimental parameters set in Appendix A Table A1, robotic ultrasonic surface strengthening is carried out in each area, and the experimental results are the surface roughness reduction value and surface hardness value of the workpiece after strengthening. The surface roughness is measured with a white light interferometer (MicroProf WLI). The surface hardness is calculated by the average hardness value measured using a Richter hardness tester at five test points in each test area.

### 4.2. Analysis of Surface Strengthening Quality

The data in Table A1 are fitted using quadratic multiple regression, as shown in Equations (10) and (11), and the three-dimensional response surface graphs and contour maps of the response of three factors to surface roughness and surface hardness are obtained. The interaction between different variables can be reflected through the response surface graphs and contour maps, from which the optimal value range of each factor can be determined intuitively.

The quadratic polynomial regression equations for surface roughness Δ*Ra* and hardness *H* are:

Δ*Ra* = 1.85 + 0.25 A − 0.17 B + 0.044 C − 0.26 A^2^ − 0.13 B^2^ − 0.07 C^2^ + 0.15 A × B − 0.03 A × C − 0.12 B × C (10)

*H* = 669 + 11.26 A − 6.34 B + 1.24 C − 10.62 A^2^ − 4.79 B^2^ − 2.08 C^2^ + 3.15 A × B+ 0.03 A × C − 2.48 B × C (11)

#### 4.2.1. Surface Roughness Response Analysis

**Figure A1:**
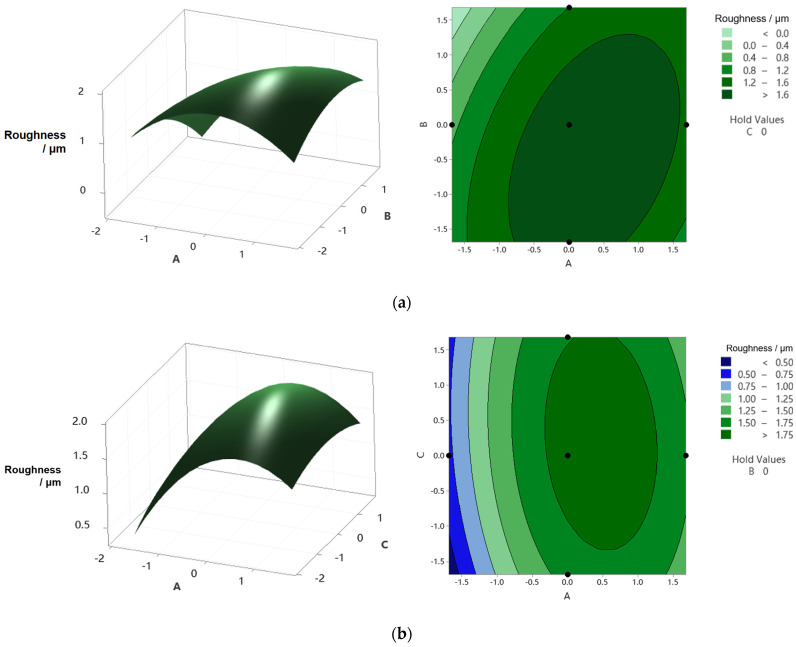

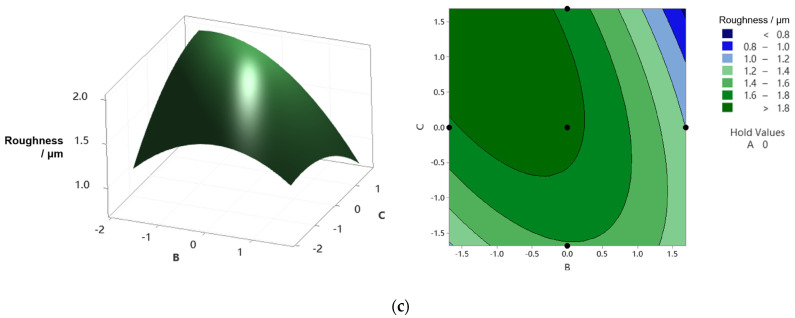
Surface graphs and contour maps of surface roughness considering the interaction among factors. (**a**) Surface roughness vs. factors A, B. (**b**) Surface roughness vs. factors A, C. (**c**) Surface roughness vs. factors B, C.

Figure A1 shows the response surface graphs and contour maps of surface roughness considering the interaction among factors. As shown in Figure A1a, the increase in feed rate can significantly reduce the surface roughness at the lower level of contact force, but at the higher level, the feed rate has little effect on the surface roughness. As shown in Figure A1b, the influence of contact force on surface roughness is greater at a low level and less at a high level. The repetition times have little influence on the surface roughness as a whole, and the interaction between the two factors is not significant. Figure A1c shows that the lower the feed rate level and the higher the repetition times, the greater the reduction in surface roughness.

By analyzing the above situation, it is attributed to the elastic–plastic deformation produced at about 25 N completely covering the blade surface, and the continuous increase in contact force will lead to excessive plastic deformation and affect the machining quality. The feed rate determines the impact frequency per strengthening path, and the higher the impact frequency, the higher the degree of strengthening. When the strengthening degree is high enough, it is difficult to further improve the surface roughness by increasing the impact frequency. The impact frequency and coverage rate per unit strengthening area are also determined by repetition times, so its influence on surface roughness is similar to that of feed rate to a certain extent. However, when the impact frequency is low, increasing repetition times can obviously improve the coverage rate and enhance the strengthening quality.

#### 4.2.2. Surface Hardness Response Analysis

**Figure A2:**
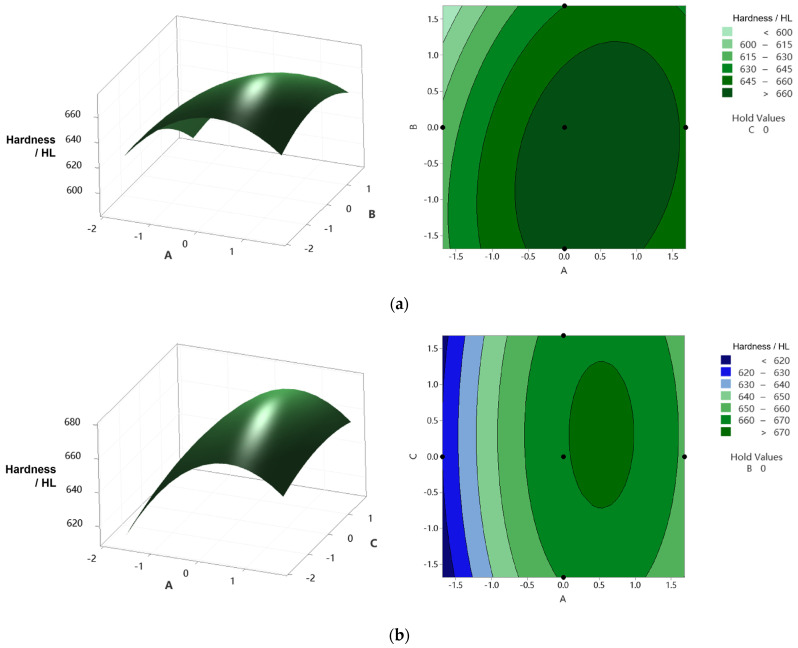

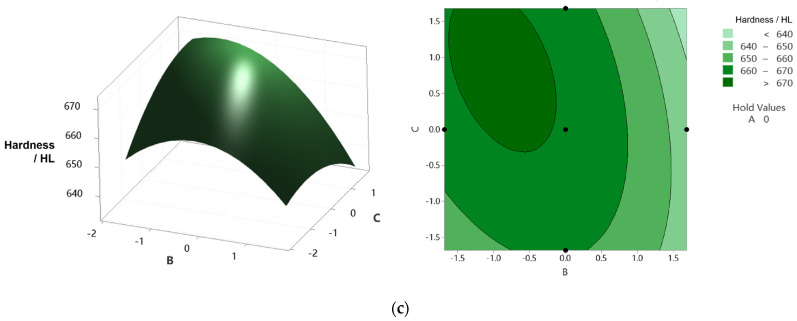
Surface graphs and contour maps of surface hardness considering the interaction among factors. (**a**) Surface hardness vs. factors A, B. (**b**) Surface hardness vs. factors A, C. (**c**) Surface hardness vs. factors B, C.

Figure A2 shows the response surface graphs and contour maps of surface hardness considering the interaction among factors.

As shown in Figure A2a, the influence of feed rate on surface hardness is significant when the contact force is low. Figure A2b reflects the influence of contact force and repetition times on surface hardness, which is similar to the influence of these two factors on surface roughness. As shown in Figure A2c, the influence of feed rate on surface hardness is more significant at high repetitions than at low repetitions.

By analyzing the above situation, the main reason that affected the surface hardness is the degree of plastic deformation that occurs on the blade surface. The decrease in feed rate and the increase in contact force and repetition times obviously enhance the degree of plastic deformation on the blade surface. The increase in plastic deformation makes the degree of work hardening higher, and the surface hardness also increases. However, when the three factors are set too high, it will lead to over-machining, which will easily lead to fatigue microcracks in the strengthened area and affect the surface quality. In addition, when the degree of work hardening is high enough, the low ultrasonic impact strength is not enough to enhance the hardness of the blade surface, so there will be a situation where a certain factor has little influence effect on the surface hardness with a certain parameter combination.

#### 4.2.3. Response Optimization

**Figure 10:**
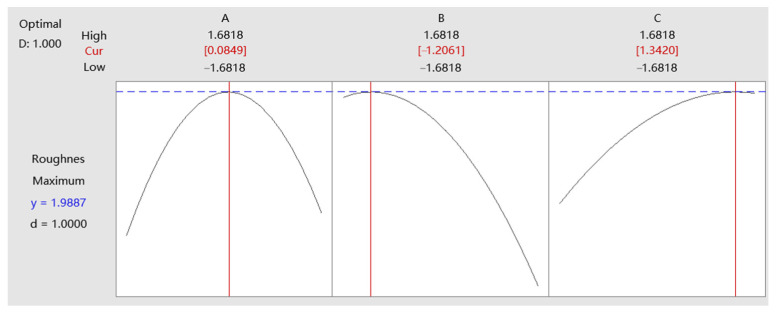
Response optimization of surface roughness.

**Figure 11:**
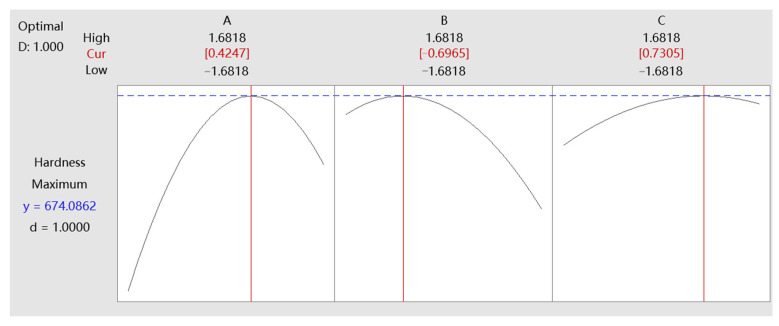
Response optimization of surface hardness.

Figure 10 and Figure 11 show the response optimization curves of contact force, feed rate, and repetition times to surface roughness and hardness, respectively, from which the combination of the optimal parameter levels of the three factors can be obtained.

When the maximum value of roughness response *y* is 1.9887, the optimal levels of the three factors are A = 0.0849, B = −1.2061, and C = 1.3420. This setting can minimize the surface roughness. When the maximum value of hardness response *y* is 674.0862, the optimal levels of the three factors are A = 0.4247, B = −0.6965, and C = 0.7305. This setting will help to obtain the best surface hardness. The D and d in the figures represent desirability, both of which are 1. Individual desirability (d) evaluates how the settings optimize a single response, and composite desirability (D) evaluates how the settings optimize a set of responses overall. Desirability has a range of 0 to 1, and the closer to 1, the better the optimal factors’ levels are set to meet the response target, so the desirability of this design indicates the settings can achieve favorable results for all responses as a whole. After data conversion, the actual optimal parameter values for the surface roughness are 25.4 N (contact force), 1.4 mm/s (feed rate), and four (repetition times), and the actual optimal parameter values for the surface hardness are: 27.1 N (contact force), 1.7 mm/s (feed rate), and four (repetition times). The optimization results of machining parameters provide a basis for selecting parameters for the industry robot to carry out ultrasonic strengthening of blade surfaces.

## 5. Quality Evaluation of Robotic Ultrasonic Strengthening of Titanium Alloy Aviation Blades

Based on the above research on contact force control, to verify the overall performance of robotic ultrasonic strengthening of the aviation blade surface, half of the blade surface is selected to conduct a contrast experiment of robotic ultrasonic surface strengthening experiment. The contact force is 25 N, the feed rate is 1.5 mm/s, and the experiment is repeated four times. The robotic strengthening path is planned, and ultrasonic parameters are set according to the previous study [13].

**Figure 12:**
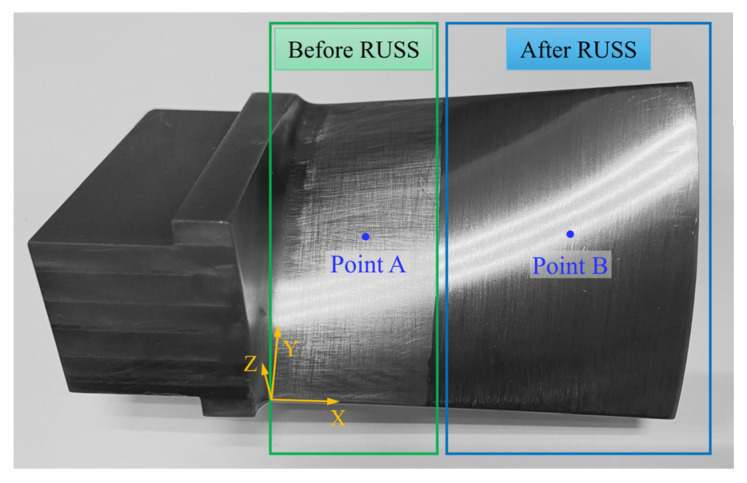
Aviation blade surface before and after robotic ultrasonic surface strengthening (RUSS).

The blade surface after robotic ultrasonic surface strengthening is shown in Figure 12. From this figure, it can be intuitively observed that the blade surface quality is significantly improved. In contrast with the other half of the surface that has not been strengthened, the scratches and cutting textures on the previous surface disappear, and the surface becomes smooth and uniform.

**Figure 13:**
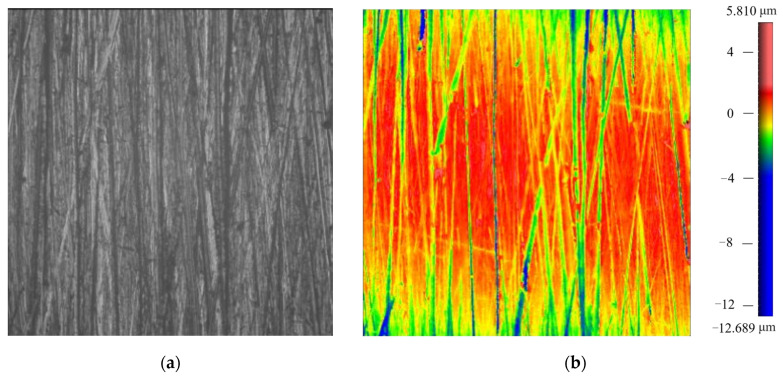
Surface micrograph of the aviation blade before robotic ultrasonic surface strengthening. (**a**) Micrograph 1 mm × 1 mm. (**b**) Surface morphology 1 mm × 1 mm.

**Figure 14:**
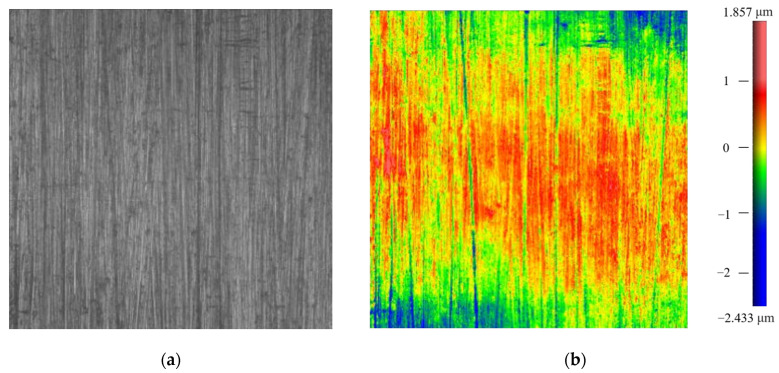
Surface micrograph of the aviation blade after robotic ultrasonic surface strengthening. (**a**) Micrograph 1 mm × 1 mm. (**b**) Surface morphology 1 mm × 1 mm.

The surface micrographs of the blade before and after strengthening are shown in Figure 13 and Figure 14. It can be observed from the micrographs that the strengthening texture with a regular stripe shape is formed on the surface, which is evenly distributed. There are no obvious over-strengthened and lack-strengthened areas, so the previously rough surface quality is well-improved. After measurement, the surface roughness of the blade is reduced from Ra 3.0 μm to about Ra 0.9 μm, and the surface hardness is increased from 582 HL to about 680 HL.

**Figure 15:**
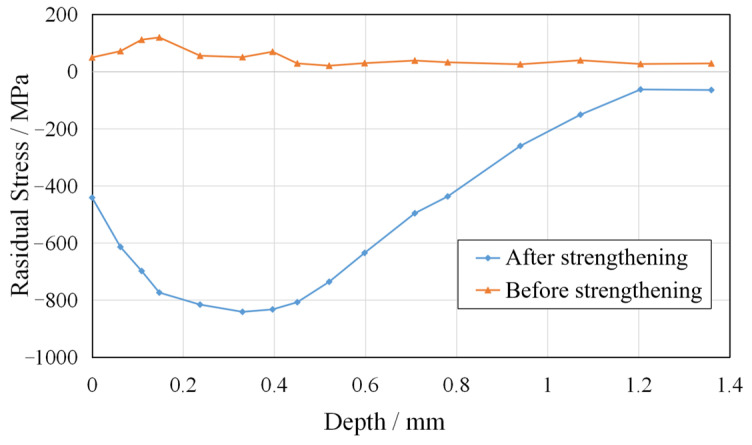
Distribution of residual stress (*σ_xx_*) on the blade surface.

Ultrasonic surface strengthening can produce a residual compressive stress layer on the workpiece surface, which has a significant effect on improving the wear resistance and fatigue resistance of the workpiece. *σ_xx_* is measured to analyze the residual stress on the blade surface. Points A and B in Figure 12 are the measurement positions, located in the center of their respective regions. The residual stress is measured using the X-ray diffraction method. As shown in Figure 15, after robotic ultrasonic strengthening, the residual tensile stress on the blade surface is converted into a larger residual compressive stress. The maximum residual compressive stress on the blade surface can reach 841 MPa, and the depth of the compressive stress layer is close to 1.2 mm. Therefore, after robotic ultrasonic strengthening, the surface of the titanium alloy blade can have a deep compressive stress layer with large residual compressive stress, which effectively improves the resistance to fatigue crack propagation of the blade and greatly prolongs its service life.

## 6. Conclusions

This paper proposes an intelligent compliance control method for robotic ultrasonic strengthening of the aviation blade surface and designs the RSM experimental method to study the interaction between the main process factors on the surface roughness and surface hardness of the blade. The contributions and conclusions are summarized below:

(1). The parallel fuzzy PID control method possesses the capability to eliminate steady-state error in the fuzzy control process, thereby enhancing the dynamic response performance of the end-effector system. The inclusion of the fuzzy link endows the system with robust adaptability and anti-interference ability, effectively suppressing system overshoot. Meanwhile, the PID link fulfills the role of accurately tracking the target value without static error. The compliance controller is adaptive and intelligent, and the regulation performance and tracking robustness of the system are improved. The compliance control strategy can realize the control optimization of the compliance device, thereby effectively improving the static and dynamic characteristics of the compliance control system.
(2). The optimal combination of machining parameters of robotic ultrasonic surface strengthening is obtained from the RSM experiments. The experimental results indicate that appropriately increasing the levels of the three factors can enhance the surface quality of aviation blades. However, it is crucial to note that excessively high levels may lead to over-processing and consequent degradation in surface quality. Using response surface analysis, the interactive effects among various factors on surface strengthening performance are clearly demonstrated, providing valuable guidance for determining the optimal parameter range in actual strengthening processes.
(3). The blade strengthening experiment verifies that the compliance control method can maintain the constant output of the contact force and significantly improve the surface quality of the blade. Table 3 shows the comparative data before and after strengthening. After robotic ultrasonic strengthening, the surface roughness of the blade is reduced from Ra 3.0 μm to about Ra 0.9 μm, and the surface hardness is increased from 582 HL to about 680 HL. The maximum residual compressive stress on the blade surface can reach 841 MPa, and the depth of the compressive stress layer is close to 1.2 mm. This compressive stress layer substantially enhances the wear resistance and fatigue resistance of the blade, consequently reducing fatigue-induced damage.

## Figures and Tables

**Table 1:** Fuzzy control rule table of contact force.

Fuzzy Control Rule Table												
*u*		*e*										
		NVB	NB	NM	NS	NVS	ZO	PVS	PS	PM	PB	PVB
***ec***	**NVB**	NVB	NVB	NB	NB	NM	NM	NS	NS	NVS	NVS	ZO
	**NB**	NVB	NB	NB	NM	NM	NS	NS	NVS	NVS	ZO	PVS
	**NM**	NB	NB	NM	NM	NS	NS	NVS	NVS	ZO	PVS	PVS
	**NS**	NB	NM	NM	NS	NS	NVS	NVS	ZO	PVS	PVS	PS
	**NVS**	NM	NM	NS	NS	NVS	NVS	ZO	PVS	PVS	PS	PS
	**ZO**	NM	NS	NS	NVS	NVS	ZO	PVS	PVS	PS	PS	PM
	**PVS**	NS	NS	NVS	NVS	ZO	PVS	PVS	PS	PS	PM	PM
	**PS**	NS	NVS	NVS	ZO	PVS	PVS	PS	PS	PM	PM	PB
	**PM**	NVS	NVS	ZO	PVS	PVS	PS	PS	PM	PM	PB	PB
	**PB**	NVS	ZO	PVS	PVS	PS	PS	PM	PM	PB	PB	PVB
	**PVB**	ZO	PVS	PVS	PS	PS	PM	PM	PB	PB	PVB	PVB

**Table 2:** Levels and coded symbols of factors for the experiment.

Factor	Coded Symbol	Unit	Factor Level				
			−α	−1	0	1	+α
Contact force	A	N	16.6	20	25	30	33.4
Feed rate	B	mm/s	1.16	1.5	2	2.5	2.84
Repetitions	C	\	1	2	3	4	5

**Table 3:** The comparative data before and after robotic ultrasonic surface strengthening.

	Surface Roughness	Surface Hardness	Maximum Residual Compressive Stress	Compressive Stress Layer
Before RUSS	Ra 3.0 μm	582 HL	\	\
After RUSS	Ra 0.9 μm	680 HL	841 MPa	1.2 mm

## Data Availability

The data of this research are being used for further extended research and can be made available in due course.

## Appendix A

**Table A1:** RSM design table and experimental results.

Run	A	B	C	ΔRa (μm)	Hardness (HL)
1	−1	−1	−1	1.33135	648.522
2	1	−1	−1	1.37933	655.673
3	−1	1	−1	0.80361	629.671
4	1	1	−1	1.61921	660.006
5	−1	−1	1	1.62784	648.231
6	1	−1	1	1.74155	666.108
7	−1	1	1	0.81914	630.042
8	1	1	1	1.33147	649.905
9	−α	0	0	0.61770	619.800
10	α	0	0	1.78113	666.507
11	0	−α	0	1.79912	670.840
12	0	α	0	1.33135	648.450
13	0	0	−α	1.67918	662.414
14	0	0	α	1.80524	672.222
15	0	0	0	1.98000	673.000
16	0	0	0	1.88908	670.840
17	0	0	0	1.78713	666.747
18	0	0	0	1.82311	668.192
19	0	0	0	1.79912	667.229
